# A38 EXPLORING HOW BOWEL PREPARATION CAN AFFECT INFLAMMATORY BOWEL DISEASE VIA THE GUT MICROBIOTA

**DOI:** 10.1093/jcag/gwad061.038

**Published:** 2024-02-14

**Authors:** C Clayton, K Ng, C Tropini

**Affiliations:** Microbiology & Immunology, The University of British Columbia, Vancouver, BC, Canada; Microbiology & Immunology, The University of British Columbia, Vancouver, BC, Canada; Microbiology & Immunology, The University of British Columbia, Vancouver, BC, Canada

## Abstract

**Background:**

Inflammatory bowel disease (IBD) is a debilitating disorder that targets the gastrointestinal (GI) tract. Although its causes remain unknown, recent studies have identified changes to the gut microbiota associated with IBD. While most gut bacteria are essential for GI health, pathobionts are bacteria that are prevalent in IBD patients and that can act as pathogens and induce inflammation. IBD patients undergo routine endoscopies which require the administration of laxative-based bowel prep to clear out the luminal contents of the GI for the endoscope. It has been found that after bowel prep some IBD patients experience inflammatory flareups. IBD patients may experience adverse reactions following bowel prep, including increased inflammation, emergency room visits, and medication adjustments. Importantly, bowel prep perturbs the gut microbiota, depleting beneficial microorganisms, while allowing pathobiont strains to thrive, which could be the cause for worsened symptoms post-bowel prep in some patients.

**Aims:**

We hypothesize that the altered intestinal microenvironment during bowel prep causes commensal bacteria depletion and favours osmotolerant pathogenic species that lead to increased inflammation in two systems: 1) in a model disease-causing microorganism, *Salmonella enterica* 2) in an IBD microbiota.

**Methods:**

To investigate pathogen expansion after bowel prep a *Salmonella* mouse model was established. Microbiota changes were determined by 16S rRNA sequencing and spot plating. Changes to the gut environment and the mechanism for pathogen colonization were characterized using *Salmonella* mutants and confocal imaging. To identify changes to the IBD microbiota, a humanized mouse model was established, and microbiota changes were investigated as done in the *Salmonella* model. IBD-associated pathobiont growth was also characterized in *in vitro* conditions that were identified in our *in vivo* model.

**Results:**

We have demonstrated that bowel prep increases GI osmolality and leads to increased *Salmonella* colonization in the gut and systemic organs following bowel prep unlike mice treated with vehicle, supporting our hypothesis. We then explored the effects of bowel prep in a humanized mouse model of IBD, which showed increased translocation of bacteria from the gut to internal organs post-prep. Additionally, IBD-associated pathobionts were able to grow much greater than commensal strains highlighting that IBD pathobionts can persist in the gut after bowel prep.

**Conclusions:**

Our study highlights that bowel prep disrupts the gut microbiota and the intestinal environment allowing for pathogen colonization and bacterial translocation. Therefore, bacterial translocation could provide mechanistic insight for inflammatory flareups following bowel prep. Ultimately, our research underscores the importance of the gut environment in facilitating pathobiont exacerbation of IBD.

**Funding Agencies:**

CIHR

